# Imaging Biomarkers in Prostate Stereotactic Body Radiotherapy: A Review and Clinical Trial Protocol

**DOI:** 10.3389/fonc.2022.863848

**Published:** 2022-04-13

**Authors:** Wei Liu, Andrew Loblaw, David Laidley, Hatim Fakir, Lucas Mendez, Melanie Davidson, Zahra Kassam, Ting-Yim Lee, Aaron Ward, Jonathan Thiessen, Jane Bayani, John Conyngham, Laura Bailey, Joseph D. Andrews, Glenn Bauman

**Affiliations:** ^1^ Department of Oncology, Division of Radiation Oncology, London Health Sciences Centre and Western University, London, ON, Canada; ^2^ Department of Radiation Oncology, Odette Cancer Center, Sunnybrook Health Sciences Centre and Department of Radiation Oncology, University of Toronto, Toronto, ON, Canada; ^3^ Division of Nuclear Medicine, St. Joseph’s Health Centre and Western University, London, ON, Canada; ^4^ Department of Oncology and Department of Medical Biophysics, London Health Sciences Centre and Western University, London, ON, Canada; ^5^ Department of Medical Imaging, St. Joseph’s Health Care and Western University, London, ON, Canada; ^6^ Department of Medical Biophysics, Western University and Lawson Health Research Institute, London, ON, Canada; ^7^ Ontario Institute for Cancer Research and Department of Laboratory Medicine and Pathobiology, University of Toronto, Toronto, ON, Canada; ^8^ Patient Partner, London, ON, Canada; ^9^ Clinical Research Unit, London Regional Cancer Program, London, ON, Canada

**Keywords:** SBRT, prostate cancer, PSMA PET, MRI, stereotactic, ultrahypofractionated

## Abstract

Advances in imaging have changed prostate radiotherapy through improved biochemical control from focal boost and improved detection of recurrence. These advances are reviewed in the context of prostate stereotactic body radiation therapy (SBRT) and the ARGOS/CLIMBER trial protocol. ARGOS/CLIMBER will evaluate 1) the safety and feasibility of SBRT with focal boost guided by multiparametric MRI (mpMRI) and ^18^F-PSMA-1007 PET and 2) imaging and laboratory biomarkers for response to SBRT. To date, response to prostate SBRT is most commonly evaluated using the Phoenix Criteria for biochemical failure. The drawbacks of this approach include lack of lesion identification, a high false-positive rate, and delay in identifying treatment failure. Patients in ARGOS/CLIMBER will receive dynamic ^18^F-PSMA-1007 PET and mpMRI prior to SBRT for treatment planning and at 6 and 24 months after SBRT to assess response. Imaging findings will be correlated with prostate-specific antigen (PSA) and biopsy results, with the goal of early, non-invasive, and accurate identification of treatment failure.

## Introduction and Background

External beam radiation therapy (EBRT) is a primary treatment modality for men with intermediate and high-risk prostate cancer. Conventional treatments are typically with fractions of 1.8–2.0 Gray (Gy)/day over a treatment duration of up to 8 weeks (70–80 Gy in 35–40 fractions). Biochemical failure (BF), as defined by the Phoenix Criteria (prostate-specific antigen [PSA] rise by 2 ng/ml or more above nadir PSA) (1), occurs in up to 35% of treated patients treated with standard EBRT by 10 years (2). Recent advances in image guidance and dose delivery have enabled new forms of EBRT, including stereotactic body radiation therapy (SBRT) (3) and focal intra-prostatic boost (4–6).

After radiation therapy, local recurrence occurs primarily at the sites of macroscopic dominant intraprostatic lesions (DILs) (7, 8). Comprehensive planning studies suggest that focal EBRT boost to DILs is dosimetrically feasible for a wide range of dose fractionations without exceeding normal tissue tolerances (9, 10). Most studies have used multiparametric MRI (mpMRI) to identify DILs in focal prostate radiation therapy (**Table 1**). The randomized controlled clinical trial FLAME showed that focal boost to DILs using standard fractionations improves the 5-year biochemical progression-free survival (bPFS) with acceptable toxicity (5, 12). DELINEATE, a single-center prospective phase II multicohort study, also confirmed the feasibility of DIL boost with standard and moderate fractionations with rectal and genitourinary (GU) toxicity comparable to contemporary series without intraprostatic boost (11). The safety and feasibility of DIL boost in extreme hypofractionation (five fractions) were validated in the Phase II 5STAR and Hypo-Flame trials (4, 6). These trials showed that toxicity for DIL boost with extreme hypofractionation compares to toxicity without boost and was lower than toxicity in the FLAME trial. Even with focal boost, however, intra-prostatic failure may be seen as a site of failure (13). Early detection of local recurrence after radiotherapy enables deployment of potentially curative salvage therapies (14, 15).

**Table 1:** Selected prospective evidence for focal intra-prostatic boost.

Trial	Trial type	Groups	Number of patients in analysis	Dose/fractionation to prostate	Dose/fractionation to pelvic nodes	Boost volume definition	Dose/fractionation to boost volume	Primary endpoint result
FLAME (5)	Multicenter RCT	Prostate RT ± GTV boost	571 total	77 Gy/35	n/a	GTV on mpMRI	Up to 95 Gy/35	Improved 5-year biochemical DFS in boost arm (92% vs. 85%)
DELINEATE (11)	Prospective single-center multi-cohort trial	Cohorts A (standard fractionation) and B (moderately hypofractionated)	105 total	Cohort A: 74 Gy/37 Cohort B: 60 Gy/20	n/a	GTV on mpMRI plus 2-mm expansion, excluding the urethra	Cohort A: up to 82 Gy/37 Cohort B: up to 67 Gy/20	Grade 2+ late rectal toxicity at 1 year was 4% for Cohort A and 8% for Cohort B
Hypo-FLAME (4)	Prospective multicenter single-arm trial	Single cohort	100	35 Gy/5 delivered weekly over 29 days	n/a	GTV on mpMRI	Up to 50 Gy/5	Acute grade 2+ GI toxicity 5%, acute grade 2+ GU toxicity 34%
5STAR (6)	Prospective single-center single arm trial	Single cohort	30	35 Gy/5 delivered weekly over 29 days	25 Gy/5	GTV on mpMRI	Up to 50 Gy/5	Acute grade 2+ GI toxicity 5%, acute grade 2+ GU toxicity 20%

RCT, randomized controlled trial; GTV, gross tumor volume; mpMRI, multiparametric MRI; DFS, disease-free survival; GU, genitourinary; n/a, not applicable.

Multiple studies have evaluated the boost of DILs using mpMRI for target delineation. However, mpMRI can miss some intraprostatic lesions or significantly underestimate lesion size (16). Prostate-specific membrane antigen (PSMA)-targeted positron emission tomography (PET) complements mpMRI and improves the detection and characterization of intraprostatic cancer and nodal disease in the primary setting (17–20). As such, it may improve oncologic outcomes through more accurate delineation of focal boost volumes (17, 18, 21). Additionally, PSMA PET provides better distant staging and can identify extra-prostatic extension, especially among men with higher risk disease (22). ^68^Ga-PSMA-11 is the most widely studied and ^18^F-DCFPyL is the next most commonly studied PSMA radioligand (23, 24). The advantages of fluorinated compounds like ^18^F-DCFPyL compared to gallium-based compounds like ^68^Ga-PSMA-11 include improved spatial resolution and a longer half-life, which allows for centralized production and transportation to remote facilities (25). ^18^F-PSMA-1007 is a third PSMA radioligand with a growing body of evidence. The primary advantage of ^18^F-PSMA-1007 compared to ^68^Ga-PSMA-11 and ^18^F-DCFPyL is its reduced urinary clearance, which allows for improved assessment of the pelvic region, making it especially suitable for the evaluation of DILs in the base of the prostate (26). A potential disadvantage of ^18^F-PSMA-1007 is a higher number of false-positive bone marrow lesions noted in some series (26).

In recent years, the clinical use of ^18^F-labeled PSMA-targeted compounds has significantly increased. ^18^F-DCFPyL and ^18^F-PSMA-1007 are the most clinically established ^18^F-labeled radiotracers for PSMA-targeted PET imaging (25). For instance, ^18^F-DCFPyL demonstrated high sensitivity for the detection of clinically significant intraprostatic tumors and biochemically recurrent prostate cancer, in addition to a high potential to measure total tumor burden for treatment planning (27, 28). We have demonstrated through a prospective trial of the preoperative imaging that ^18^F-DCFPyL-PET/MRI could identify DILs as verified by whole-mount pathology images (29). The performance of delineation of DILs for focal treatment could be optimized by using a 67% threshold of the maximum intra-prostatic standard uptake value (SUV) with an 8-mm margin to maximize coverage of histologically defined lesions (30).

The alternate PSMA-targeting agent, ^18^F-PSMA-1007, offers additional advantages related to the delineation of intraprostatic lesions. While ^18^F-DCFPyL is excreted by renal clearance into the urinary bladder, ^18^F-PSMA-1007 is excreted by the hepatobiliary system and therefore causes no or minimal bladder activity. A comparison of ^18^F-PSMA-1007 PET/CT with radical prostatectomy histology and mpMRI (n = 10) showed a slightly better performance than mpMRI with fewer false negatives and fewer false positives (31). A clinical comparison of [^18^F]DCFPyL and ^18^F-PSMA-1007 (n = 12) found excellent image quality and identical clinical findings. Both radiotracers were equivalent for imaging of local and metastatic prostate cancer. However, the non-urinary excretion of ^18^F-PSMA-1007 offers advantages regarding the delineation of local recurrences and lymph node metastases (32). Prive et al. evaluated ^18^F-PSMA-1007 and mpMRI and compared their histopathology for the primary staging of prostate cancer in 53 patients diagnosed with intermediate and high-risk prostate cancer. PSMA improved the detection of seminal vesicle invasion, while MRI offered a better resolution in evaluating extracapsular extension (33). The study suggested that dual imaging may improve the staging of prostate cancer. A 20% SUVmax threshold using ^18^F-PSMA-1007 was recently demonstrated to offer the best combination of sensitivity and specificity in delineating DILs, and volumes so defined accounted for approximately 21% of the total prostate volume on average (18).

Another application for advanced imaging in SBRT prostate treatment is in response assessment. To date, evaluation of success following SBRT is most commonly by biochemical means, and successful SBRT is associated with low PSA nadirs comparable to those noted with brachytherapy (34). Biochemical control is a suboptimal method to assess recurrence in patients due to a lack of spatial information, potential false positives, and delayed identification of failure based on rising PSA. First, the lack of lesion identification using PSA-based criteria alone prevents successful local or metastasis-directed salvage without the use of imaging. Given the potential toxicity of local salvage, identification of isolated local recurrence is critical (14). Secondly, the Phoenix Criteria has a false-positive rate in patients who receive SBRT. In a multi-institutional pooled analysis of over 2,000 patients who received prostate SBRT, the Phoenix Criteria was associated with a false-positive rate of 30% (35). Finally, the Phoenix Criteria occurs late. Patients who have local failure may not reach the Phoenix Criteria for years and may lose the opportunity for successful local salvage. A retrospective study showed that up to 38% of patients who received SBRT to doses of 32.5 Gy or higher in 5 fractions had a positive prostate biopsy 2 years after SBRT (36). However, just 12.5% of these patients had reached the Phoenix Criteria at the time of biopsy. Even in patients with a PSA of less than 1 ng/ml prior to biopsy, up to 25% of patients had a positive biopsy (36). Most patients with a positive 2-year biopsy would reach BF at 5 years (57% vs. 7% as compared to those with a negative biopsy), even after 35% of patients with a positive 2-year biopsy received salvage therapies. In another retrospective study, 63 patients, mostly with high-risk prostate cancer (40/63, 64%), received PSMA-targeted PET/CT for rising PSA that did not meet the Phoenix Criteria after primary conventional or moderately hypofractionated EBRT (37). Median rise above nadir PSA prior to PET was 1.2 ng/ml, and median PSA was 1.3 ng/ml. Recurrence was detected in 84% of patients (53/63). While 21/63 patients (33%) had local recurrence only, 14/63 (22%) had nodal recurrence without distant metastases, and 18/63 (18%) had distant metastases. Given the efficacy and toxicity of curative-intent local salvage treatments, improved and early identification of isolated local recurrence is needed (14, 15).

Identification of local recurrence after EBRT has been explored with timed biopsy or mpMRI after radiotherapy (38). Prostate biopsy at 2 years posttreatment has been associated with clinical endpoints such as subsequent BF and distant metastases (36, 39). However, drawbacks of biopsy include unreliable results at earlier timepoints and potential morbidity (39). “Metabolic clearance” as defined by serial MRI with spectroscopy has been associated with durable biochemical control in retrospective series of men treated with conventional external beam radiotherapy (40–43). Recently, standardized mpMRI reporting for the locally recurrent disease has been proposed but has not yet been validated in larger prospective series (44). Additionally, there is a lack of prospective studies validating posttreatment mpMRI as a predictive biomarker in larger populations and men treated with SBRT.

PSMA-targeted PET/CT in addition to mpMRI improves detection of local recurrence after EBRT (45). However, while criteria for the response have been broadly defined (46), the significance of PSMA response and correlation with clinical endpoints are not known (47). As such, longitudinal monitoring of PSMA PET/CT changes post-radiotherapy, including changes in SUVmax and other PSMA PET based metrics, should be investigated as potential non-invasive biomarkers of treatment response after SBRT to the prostate. Integration of earlier PET-based response assessment, compared to triggered restaging at the time of BF, may provide an opportunity for earlier targeted salvage, but a lack of prospective longitudinal series of men so monitored is a gap in the current evidence base (23). Indeed, reports of false-positive PET scans in previously treated patients underscore the importance of systematically characterizing the normal patterns of PSMA PET/CT changes after SBRT and their correlation with clinical endpoints (48, 49).

Beyond identifying local recurrence, determining the presence and extent of extra-prostatic recurrence has historically been challenging to determine due to the poor sensitivity of CT and bone scans. PET-based imaging potentially addresses this gap (23). A number of PET tracers have been developed for the detection of recurrent prostate cancer, including ^18^F-NaF, ^18^F-FACBC (fluciclovine), ^18^F-choline, and ^11^C-choline (50). More recently, PSMA-targeted PET has demonstrated improved detection rates as compared to previous modalities and is recommended for restaging recurrent disease (23, 24, 50, 51). Specifically, for patients with BF after primary radiotherapy, a prospective trial showed that compared to conventional imaging, PSMA-targeted PET/CT detected extra-prostatic recurrence in twice as many patients (39% vs. 19%) (52, 53). Furthermore, in a network meta-analysis of intra-individual imaging studies of different radiotracers, PSMA-based tracers in general, and ^18^F-PSMA-1007 specifically, were found to have superior detection rates at any site as compared to other traces, including ^18^F-FACBC, ^18^F-choline, and ^11^C-choline (54). However, the strength of these findings was tempered by the relatively small number of ^18^F-1007 patients evaluated directly. While PSMA-targeted PET/CT has increased detection rates in recurrent prostate cancer, a drawback is the risk of false-positive findings. In a prospective trial that evaluated the positive predictive value of PSMA-targeted PET/CT in patients with recurrent prostate cancer, the per-region false-positive rate based on a clinical endpoint was 8% (55).

We plan to evaluate the safety of SBRT with PSMA PET/MRI-guided focal boost in a prospective early phase trial, “PSMA MRI Guided prOstate SBRT(ARGOS).” As noted, while the advanced imaging techniques described show promise for the characterization of primary or recurrent prostate cancer, no study has prospectively and longitudinally evaluated them after primary radiotherapy to characterize expected changes in response to treatment and to non-invasively identify early treatment failure. All patients in ARGOS will enter the translational component of the study Comprehensive, Longitudinal Evaluation of Imaging Biomarkers Post Radiotherapy (CLIMBER). We will use advanced imaging analysis techniques to evaluate longitudinal changes in a comprehensive battery of anatomic and functional prostate imaging panels using ^18^F-PSMA 1007 and mpMRI acquired prior to and after SBRT. ^18^F-PSMA-1007 is chosen given its favorable pharmacokinetics profile with primarily gastrointestinal elimination, reducing tracer accumulation in the bladder and allowing better visualization of prostate and pelvic lymph nodes. The knowledge obtained from ARGOS/CLIMBER will improve the understanding of imaging changes post-prostate SBRT and will have increasing clinical importance with increasing use of these techniques. The ARGOS/CLIMBER protocol as outlined below is due to open in early 2022, with a plan for accrual of 50 men over 3 years and with follow-up of up to 5 years for clinical endpoints.

## ARGOS/CLIMBER

### Study Design

**Figure 1:**
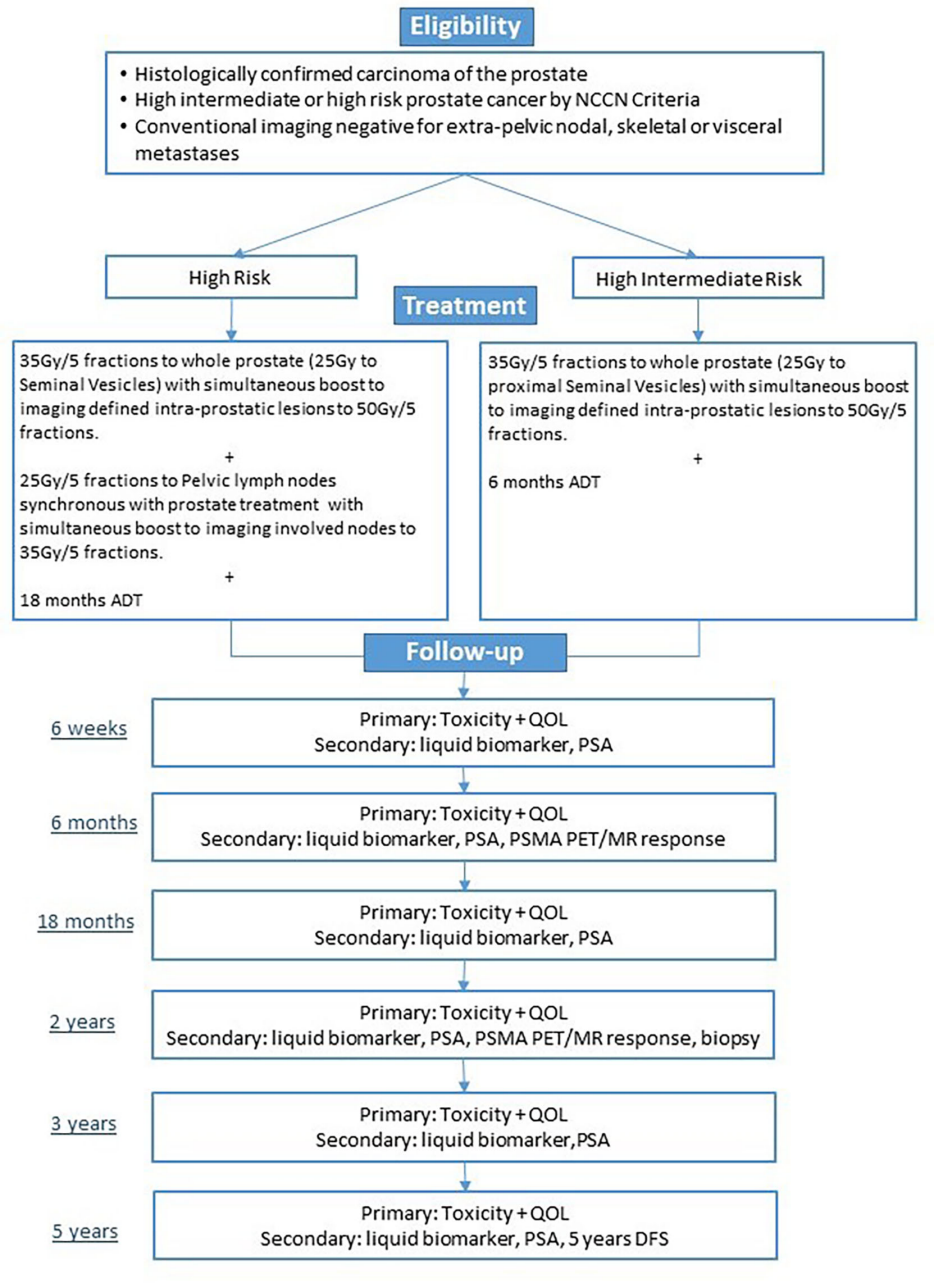
Study schema.

**Figure 2:**
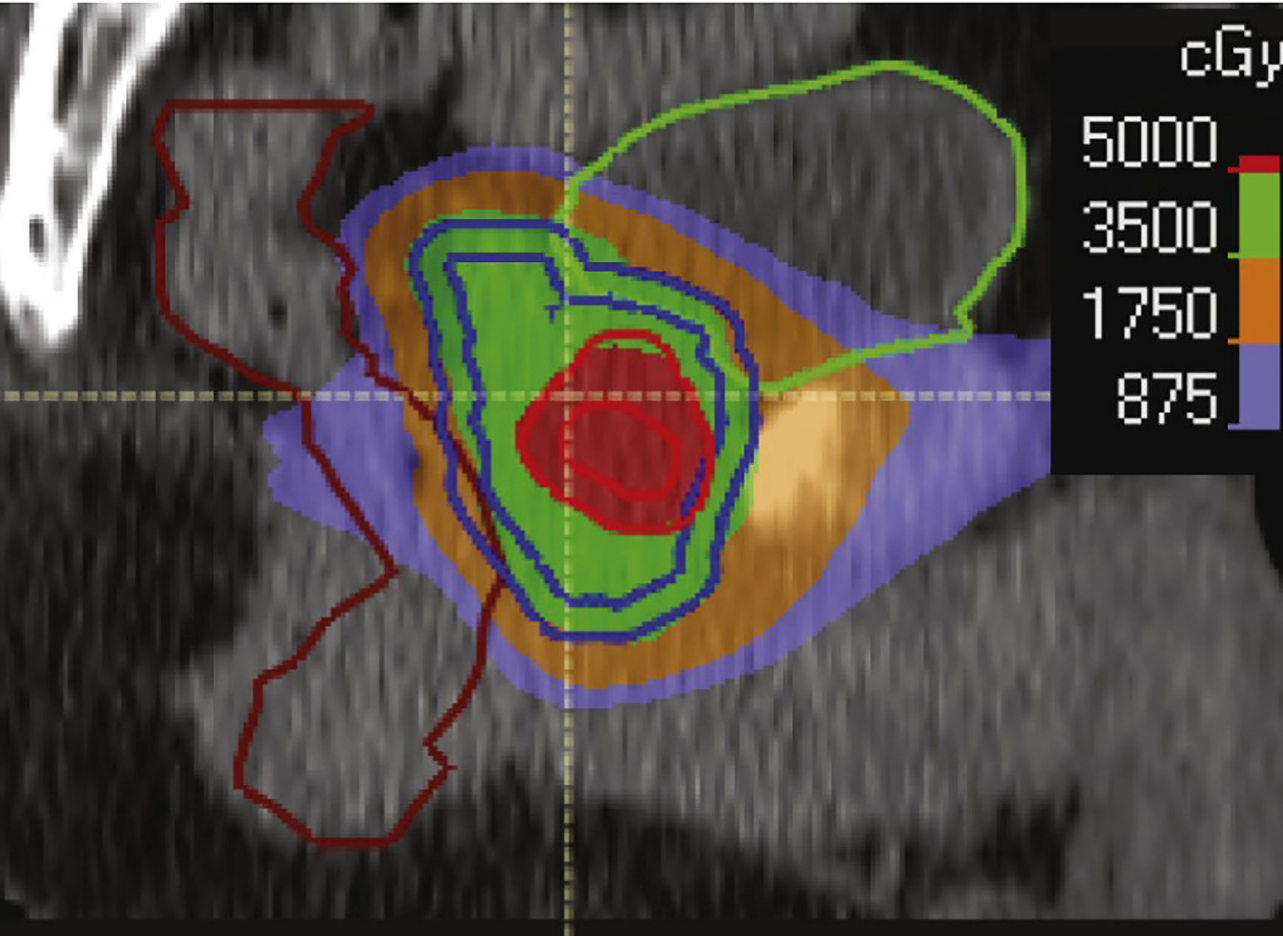
Example of focal dose escalation.

This study is (NCT05269550) a prospective single-arm trial enrolling men with National Comprehensive Cancer Network (NCCN) unfavorable intermediate-fiducial risk, high-risk, or very-high-risk prostate cancer. The study schema is provided in **Figure 1**. All men will have PSMA-targeted PET (using the PSMA-targeting ligand ^18^F-PSMA-1007) and mpMRI including T2-weighted (T2W), diffusion-weighted imaging (DWI)/apparent diffusion coefficient (ADC), and dynamic contrast-enhanced (DCE) sequences. Delineation of intra-prostatic foci of cancer (using 20% SUVmax and suspicious mpMRI appearance) and any involved regional lymph nodes (based on MI-ES score of 2 or greater or suspicious mpMRI appearance suspicious for cancer) will be performed (16, 18). Tumor delineation will be performed by fusing the PSMA PET and mpMRI with planning CT simulation images. Fiducial marker implantation for treatment guidance will be mandatory, but the use of other organs at risk protection strategies (i.e., SpaceOAR and GU-Lok) will be allowed but not mandatory. Patients will be treated with image-guided SBRT using the fiducial markers for inter- and intra-fraction motion management. The prostate will receive 35 Gy/5 fractions, and the proximal or entire seminal vesicle will receive 25 Gy/5 fractions (**Table 2**). Dose escalation to imaging-defined targets will be accomplished through a simultaneous boost technique (targeted maximum dose of 50 Gy/5 fractions to imaging-defined prostatic lesion and 35 Gy/5 fractions to imaging-defined involved nodes; see **Figure 2**). Maintaining dose to organs at risk will take precedence over boost dose targets (**Table 3**). Patients with high-risk disease or calculated nodal involvement risk of more than 15% will receive 25 Gy/5 fractions delivered to the regional lymph nodes synchronously with the prostate treatment.

**Table 2:** Target structures nomenclature and descriptions.

Name*	Description
**High intermediate risk**	
CTV_35Gy	Entire prostate including the GTVp_boost volumes
PTV_35Gy	CTV_35Gy + 3–4 mm
GTVp_boost	Intraprostatic GTV delineated as the union of mpMRI-defined PiRADS 4–5 intra-prostatic lesions with the PET-defined intra-prostatic lesions using threshold of 20% SUVmax (see text above). Where the seminal vesicle(s) are involved by PET or MRI, the involved portion will be included in the GTVp_boost volume(s)
PTVp_boost	GTVp_boost + 3–4 mm
CTV_ProxSV_25Gy	Proximal 1.0 cm of the seminal vesicles. The 1 cm of the seminal vesicles is measured superiorly from its origin at the prostate (not from the superior aspect of the prostate)
PTV_ProxSV_25Gy	CTV_ProxSV_25Gy + 4 mm
**High or Very High Risk**	
CTV_35Gy	Entire prostate including the GTVp_boost volumes
PTV_35Gy	CTV_35Gy + 3–4 mm
GTVp_boost	Intraprostatic GTV delineated as the union of mpMRI-defined PiRADS 4–5 intra-prostatic lesions with the PET-defined intra-prostatic lesions using threshold of 20% SUVmax (see text above). Where the seminal vesicle(s) are involved by PET or MRI, the involved portion will be included in the GTVp_boost volume(s)
PTVp_boost	GTVp_boost + 3–4 mm
CTV_ SV_25Gy	Entire seminal vesicle volume
PTV_SV_25Gy	CTV_SV_25Gy + 6 mm
CTVn_25Gy	Pelvic lymph nodes. To be contoured according to the NRG guidelines [51] to encompass a 0.7-cm radial expansion around the external iliac, internal iliac vessels, and obturator and presacral spaces
PTVn_25Gy	CTVn_25Gy + 6 mm
GTVn_boost	Positive pelvic lymph nodes delineated on PET/MRI as MI-ES 2 or higher
PTVn_boost	GTVn_boost + 6 mm

*GTV, gross tumor volume; CTV, clinical target volume; PTV, planning target volume; mpMRI, multiparametric MRI.

**Table 3:** Dose constraints.

Structures and dose constraints (acceptable deviations)			
**Rectum**	V20Gy ≤ 50% (optimal) V28Gy ≤ 15% (20%) V32Gy ≤ 10% (15%) V35Gy ≤ 2 cc (4 cc) V38Gy ≤ 1 cc Dmax ≤ 40.6 Gy	**Bladder**	V20Gy ≤ 50% (optimal) V28Gy ≤ 15% (20%) V32Gy ≤ 10% (15%) V38Gy ≤ 6 cc V39.5Gy ≤ 2 cc
**Rectum_PRV**	Dmax ≤ 45 Gy	**Bladder_PRV**	Dmax ≤ 46 Gy (optimal)
**Urethra_PRV**	Dmax ≤ 52 Gy D10% ≤ 47.2 Gy D50% ≤ 42 Gy (optimal)	**Penile Bulb**	V20Gy ≤ 40% (90%) V35Gy ≤ 4%
**Bowel_Small**	V25Gy ≤ 20 cc (40cc) V30Gy ≤ 2 cc Dmax ≤ 3 5Gy	**Bowel_Large**	V25Gy ≤ 20 cc (40 cc) Dmax ≤ 38 Gy
**Femur_R and Femur_L**	V28Gy ≤ 5%		

The primary endpoints of the trial will be 6-week and 6-month gastrointestinal (GI) and GU toxicity using the Common Terminology Criteria for Adverse Events version 5.0 (CTCAE v5.0). While other prospective trials have confirmed the safety of an mpMRI-defined intra-prostatic boost with external beam radiotherapy (4, 6), the proposed boost volume in ARGOS/CLIMBER will be based on mpMRI and PSMA PET-targeted PET (17), and thus there is a need to demonstrate the safety of such a multi-modality defined boost volume. Secondary endpoints include Quality of life measured by the Expanded Prostate Cancer Index Composite (EPIC-26) questionnaires and 5-year disease-free survival (DFS) as a composite of BF, patient death, or development of clinical metastases or institution of salvage ADT.

All grade 3 or higher toxicity will be reported to the principal investigator. An independent data and safety monitoring board (IDSMB) will perform a formal interim analysis for safety and toxicity when half of the patients have been accrued or after 1 year, whichever comes first. The study will be discontinued if the projected rate of grade 3 or higher urinary or bowel toxicity exceeds 30%. IDSMB will meet at least annually to review trial data.

Unique to the ARGOS/CLIMBER protocol is the integration of longitudinal imaging with PSMA-directed PET and mpMRI pretreatment and posttreatment. Serial PSMA PET/MR images will be collected at baseline, 6 months, and 2 years to characterize the imaging response of prostate cancer to treatment and potentially identify imaging biomarkers (including pharmacokinetics, radiomics, and quantitative PET and mpMRI metrics) that predict for 5-year DFS (**Table 4**). Additionally, baseline collection of diagnostic tissue biopsy samples and serial collection of blood and urine over multiple time points (baseline, 6 months, 1 year, and 2 years post-SBRT) will be performed for correlative biologic biomarker analyses with imaging changes, DFS, and toxicity posttreatment. Analysis of prostate biopsy at baseline and 2 years will allow for correlation of histopathology with PSMA PET/MR images. We will investigate whether a negative posttreatment PSMA PET/MRI is correlated with a negative 2-year posttreatment biopsy and long-term disease control (36, 39). We also plan to examine novel clinical prognostic biomarkers (i.e., absolute percentage of Gleason Pattern 4 on biopsy and 4-year PSA response rate) and their correlation with imaging findings and 5-year DFS.

**Table 4:** Schedule of events.

Event	Weeks (week 0 is start of RT)										Q6mo (30–60mo)
	−3	−2	0	1 EOT	3	8 (6 weeks post-RT)	6 months post-RT	12 months post-RT	18 months post-RT	24 months post-RT	
	V1	V2	V3	V4	V5	V6	V7	V8	V9	V10	V11–15
Start alpha antagonists, simethicone	x										
Fiducial marker insertion		x									
Simulation and planning		x									
Treatment (5 fractions q2d, 10–12 days)			x	x							
CTCAE v5.0	x		x	x	x	x	x	x	x	x	x
EPIC-26 questionnaires	x		x	x		x	x	x	x	x	x
PSA and testosterone	x					x	x	x	x	x	
PSMA PET/MRI		x					x			x	
Liquid biomarker collection		x				x	x	x		x	
Transperineal biopsy		x								x	

CTCAE v5.0, Common Terminology Criteria for Adverse Events version 5.0; PSA, prostate-specific antigen; PSMA, prostate-specific membrane antigen.

### PET Imaging Acquisition

Integrated PSMA PET/MRI is preferred with the goal of achieving co-registered PSMA PET and MR images with high spatial fidelity for planning and assessing response to treatment. PSMA PET/CT plus mpMRI are also allowed within the protocol if there is an unavailability of a PET/MRI scanner. We have previously demonstrated the value of early dynamic PET imaging in the identification of intra-prostatic lesions and will incorporate both dynamic and delayed PET imaging (56).

The evening before each PSMA PET examination, patients will be asked to take 30 ml of milk of magnesia, an over-the-counter laxative, which will be provided. Patients should be NPO overnight prior to the exam (~12 h). The bladder should be comfortably full and the rectum as empty as possible prior to image acquisition.

For dynamic PET imaging, the participants will be injected with 3–4 MBq/kg (up to a maximum 400 MBq) of ^18^F-PSMA-1007. Dynamic PET acquisitions will start immediately prior to ^18^F-PSMA 1007 injection and will be acquired simultaneously with the cross-sectional pelvic MR images (for PET/MRI) or CT images (for PET/CT). Dynamic PET acquisition will cover the whole prostate up to the iliac crest. An image-derived arterial time–activity curve required for kinetic analysis of dynamic PET data will be acquired from an internal iliac artery to generate parametric maps. Starting at the injection of ^18^F-PSMA-1007 as a bolus into an antecubital vein, the dynamic PET scan will be acquired over 22 min with seven framing intervals: 10, 20, 40, 60, and 180 s. Early time standardized uptake (SUV_early_) in g/ml will be measured as the average of the last four dynamic PET volumes (10–22 min post-injection). The acquired dynamic volumes will be analyzed to generate parametric maps of the whole prostate, including influx rate constant (K_1_), efflux rate constant (k_2_), binding rate constant (k_3_), dissociation rate constant (k_4_), net uptake rate constant from plasma (K_i_), and distribution volume (DV) maps by deconvolving the arterial time–activity curve from tissue time–activity curve using a flow-modified two-tissue compartment (F2TC) model. After dynamic pelvic PET, participants will be allowed to get up and take a break/empty their bladder prior to the acquisition of late uptake PSMA-1007 PET images (60–120 min post-injection). For PET/CT, the PET images will be acquired with corresponding axial CT images obtained (for anatomic correlation and attenuation correction).

For PET/MRI, a whole-body MRI scout scan (to plan the study) and B0 homogenization using gradient enhancement (HUGE) acquisition (to correct for truncation of arms) will be acquired first. In both acquisitions, the table moves continuously for approximately 1 min as it scans the subject from head to thigh. Whole-body PET/MRI is acquired in multiple bed positions. For men of average height, 5 overlapping table positions will be used, with taller subjects requiring an additional table position. At each table position, a 5-min PET acquisition will be acquired along with simultaneous MRI consisting of MRI-based attenuation correction, coronal T2-weighted fast spin-echo with Short-TI Inversion Recovery (STIR) sequence during flat breathing, and axial Half-Fourier Acquisition Single-shot Turbo spin-Echo (HASTE) single-shot T2-weighted sequence. For thoracic and abdominal table positions, the HASTE MRI will be captured over 4 breath-holds of 14 s. If unable to do so, these can be done with flat breathing only.

### Pelvic Multiparametric MRI Acquisition

For men imaged with PET/MRI, the pelvic mpMRI will be acquired after whole-body PET/MRI on the PET/MRI scanner. For men imaged with PET/CT, mpMRI will be acquired as a separate study on a 3T magnetic resonance scanner. The bladder should be comfortably full and the rectum as empty as possible prior to the mpMRI scanning. For mpMRI scout scans, sagittal 2D T2-weighted, coronal 2D T2-weighted MRI, axial 3D T2-weighted, and 2D axial diffusion-weighted EPI will be acquired. Prior to a 3D DCE T1-weighted MRI, a radiologist or designate will administer an intravenous injection of GADOVIST^®^ 1.0 (Gadobutrol) with the MEDRAD Injection System (0.1 mmol/kg). Following DCE-MRI, whole-body late gadolinium-enhanced MRI will be acquired with T1-weighted volumetric interpolated breath-hold examination (VIBE) with fat saturation and breath-hold in thoracic and abdominal table positions.

### Primary Endpoint and Sample Size

This will be a single-phase pilot study of 50 patients with a primary endpoint of GI and GU toxicity as measured by CTCAE v5.0.

Unacceptable toxicity will be defined as acute (6 weeks) or intermediate (6 months) grade >3 GI or GU toxicity. The proposed treatment will be deemed too toxic if >30% of patients have unacceptable toxicity. This study tests the hypothesis that acute toxicity is <30% (alpha = 0.05, power = 81%, one-sided, H0: p = .30, HA: p <.30), with an assumed true proportion in this study of 15%. These calculations were done based on a Z test (normal approximation). We will test this assumption with the exact test approach if we do not meet our target accrual of 50 men or the proportion of Grade 3 toxicity is significantly less than 15% (conditions where normal approximation is not met).

Given that the proPSMA study demonstrated that 16% of men with high-risk prostate cancer had extra-prostatic disease beyond regional nodal metastases at initial staging and the fact our population will be a mix of high-intermediate and high-risk men, we will plan to enroll a total of 55 men (22). Those men with extra-prostatic spread beyond regional pelvic lymph nodes on their pretreatment PSMA PET imaging will be treated off protocol at the attending physician’s discretion.

### Secondary Endpoints

#### Quality of Life

Descriptive statistics and diagrams will be used to characterize changes in Quality of Life metrics as measured by the EPIC-26. A linear mixed model with random intercept by an individual to account for the correlation present within individuals will be used to compare pretreatment vs. posttreatment quality of life measures at multiple timepoints with the goal of tracking minimally important differences in these parameters (57).

#### Disease-Free Survival

Five-year DFS will be determined as a composite of biochemical control, patient death or development of clinical metastases, or institution of salvage ADT. DFS will be estimated with a Kaplan–Meier (KM) curve, with the 5-year estimate extracted from the KM curve.

#### Translational Imaging Endpoints

Changes in SUV metrics (SUVmax, SUVmean) within PSMA PET regions of interest (ROI) will be compared between the pre-RT PSMA PET and the 6-month post-RT PSMA PET. ROIs to be examined will include the dominant intra-prostatic lesions (DILs), the prostate as a whole, and, in the cases of men with PET-detected nodal disease, involved node ROIs. Descriptive statistics and diagrams (i.e., waterfall plots) will be used to characterize changes in SUV metrics. A linear mixed model with random intercept by an individual to account for the correlation present within individuals will be used to compare pretreatment vs. posttreatment SUV values at multiple timepoints. Overall response rates will be calculated in accordance with recent consensus guidelines (46).

Intra-prostatic mpMRI (T2W, DWI, and DCE-MRI) acquired pre-RT and 6 and 24 months post-RT will be reported by expert readers based on PI-RADS 2.1 and the complementary Prostate Imaging for Recurrence Reporting (PI-RR) system to identify intra-prostatic ROIs (44). Quantitative MRI metrics will be extracted, including ADC and pharmacokinetics parameters derived from dynamic PET and DCE-MRI. Radiomics approaches will be used to characterize the evolution of higher-level feature changes in PET and mpMRI over the course of treatment.

We will correlate changes in PET and mpMRI metrics at 6 and 24 months with 5-year DFS using linear regression models. We will also perform supervised machine learning to train support vector machines and random forest classifiers to predict response based on the pretreatment images. We will also perform a delta-radiomics analysis to predict response based on the radiomics trajectory computed from the first two time points. We will measure the performance of the classifiers using a cross-validation design, with metrics including the area under the receiver operating characteristic (ROC) curve and the error rate, false-positive rate, and false-negative rate computed at a point on the ROC curve that best balance the false-positive and false-negative rates. We will develop radiomics-based classifiers to predict 5-year DFS.

Baseline (pretreatment) and 24-month (posttreatment) tissue samples will be acquired for histopathologic correlations with PET/MR images. Specifically, baseline biopsy will provide histologic correlation for the PSMA- and mpMRI-identified dominant intra-prostatic lesions. Additionally, 24-month biopsies have been shown to correlate with long-term failure-free survival (36, 39), and rates of cancer clearance after stereotactic techniques have been shown to increase with increasing doses of radiation (58). Understanding histologic correlations and clearance of cancer from the boosted and non-boosted prostate areas will be of interest and will allow for correlation with PET/MR images to validate PET+MRI as non-invasive surrogates for identifying intra-prostatic cancer foci.

## Discussion/Conclusion

Advanced prostate imaging with mpMRI and novel PET agents has the potential to improve prostate cancer management across the disease spectrum (23). In the primary management of prostate cancer, improved imaging guidance has allowed for radiotherapy advances for prostate cancer, including prostate SBRT and focal boost (3–6, 11). Ongoing trials are evaluating SBRT with focal boost guided by mpMRI and PSMA-PET (NCT04243941, NCT04402151, and NCT04599699) (21, 59). The ARGOS/CLIMBER trial will explore the safety of SBRT with focal boost guided by mpMRI and ^18^F-PSMA-1007 PET.

In addition, advanced imaging has improved the ability to characterize patterns of disease recurrence and identify men with isolated local recurrence who may be suitable for local salvage or oligometastatic recurrence who may be eligible for metastasis-directed therapy (14, 45, 52, 60). To date, response to prostate SBRT is mostly commonly evaluated using biochemical response with the Phoenix Criteria for BF. The drawbacks of this approach include lack of lesion identification, a high false-positive rate, and delay in identifying treatment failure. An important knowledge gap is the expected evolution of imaging changes post-SBRT and whether patterns in these changes can serve as early biomarkers of disease recurrence. Patients in ARGOS/CLIMBER will receive dynamic ^18^F-PSMA-1007 PET and mpMRI prior to SBRT and at 6 and 24 months after SBRT. Imaging findings will be correlated with PSA and biopsy results, with the goal of early, non-invasive, and accurate identification of treatment failure.

## Data Availability Statement

The original contributions presented in the study are included in the article/supplementary material. Further inquiries can be directed to the corresponding author.

## Ethics Statement

The studies involving human participants were reviewed and approved by the Ontario Cancer Research Ethics Board. The patients/participants provided their written informed consent to participate in this study.

## Author Contributions

All authors contributed to the project design and manuscript drafting. All authors approved the publication of the content and agree to be accountable for all aspects of the work.

## Funding

The ARGOS/CLIMBER study is supported by the Clinical Translation Program of the Ontario Institute for Cancer Research, grant identifier P.CTP.624.

## Conflict of Interest

T-YL declares receiving royalties from GE Healthcare. JB declares receiving grants from Thermo Fisher Scientific and payments or honoraria from Thermo Fisher Scientific and NanoString Technologies, Inc., and has two pending patents. GB declares grants from the Ontario Institute for Cancer Research, Ontario Health, and Centre for Probe Development and Commercialization and industry collaboration with Siemens Healthineers and InVicro.

The remaining authors declare that the research was conducted in the absence of any commercial or financial relationships that could be construed as a potential conflict of interest.

## Publisher’s Note

All claims expressed in this article are solely those of the authors and do not necessarily represent those of their affiliated organizations, or those of the publisher, the editors and the reviewers. Any product that may be evaluated in this article, or claim that may be made by its manufacturer, is not guaranteed or endorsed by the publisher.
